# Innovation ecosystems in health and care: the Andalusian Reference Site as an example

**DOI:** 10.3389/fmed.2024.1482830

**Published:** 2024-12-02

**Authors:** A. M. Carriazo, F. Alonso-Trujillo, F. J. Vázquez-Granado, I. Túnez, M. L. Del Moral-Leal

**Affiliations:** ^1^Regional Ministry of Health and Consumer Affairs of Andalusia, Seville, Spain; ^2^Reference Site Collaborative Network, Brussels, Belgium

**Keywords:** innovation ecosystem, active and healthy living, European networks, health and care systems, Reference Site Collaborative Network

## Abstract

Innovation ecosystems foster collaboration between academia, industry, public bodies, and civil society to drive technological and social advancements. The European Innovation Partnership on Active and Healthy Aging (EIP on AHA), launched in 2012, aimed to extend healthy life years, improve healthcare efficiency, and stimulate economic growth. Reference sites (RSs) and action groups (AGs) were key components, with RSs adopting collaborative approaches to improve health outcomes. Andalusia, Spain, achieved top recognition across multiple EIP on AHA calls for its digital health strategies and strong Quadruple Helix collaboration. In 2022, Andalusia’s self-assessment using the SCIROCCO tool highlighted strengths in digital transformation and citizen empowerment. Andalusia’s innovative practices in health have contributed to regional improvements in healthcare efficiency, life expectancy, and research initiatives.

## 1 Introduction

Innovation is a central tool for the advancement of the economy and society (1). Innovation ecosystems refer to a context or environment in which technological and other innovations are favored by a set of dynamic agents following a Quadruple Helix implementation based on the design thinking innovation model (2): research/academia, industry, public bodies and healthcare providers, and civil society. Different actors merge in innovation ecosystems where each one contributes something to its construction, either with investment, ideas, or work (3). Although innovation clusters and ecosystems may vary, a common operational and organizational scheme can be found in all of them (5).

In social and health issues, innovation addresses regional needs with a coordinated, participatory, integrated, and intersectional approach. Health and care systems across Europe are challenged by demographic changes, with increased longevity as an achievement of modern societies but with more people requiring more care. European Union health and care systems, despite diversity, are crucial for Europe’s social protection and contribute to social cohesion and sustainable development.

Recognizing innovation’s role in these challenges, the European Innovation Partnership on Active and Healthy Aging (EIP on AHA) was launched in 2012 (5) within the Innovation Union policy of the European Commission (EC) and was operational till December 2020 (6), when the financial framework 2014–2020 ended. Its main aim was to increase 2 years of healthy life for European Union citizens by 2020 by improving the sustainability and efficiency of healthcare systems and generating growth and market opportunities (the triple win). The EIP on AHA had three pillars: prevention, screening, and early diagnosis; care and cure and active aging and independent living; and horizontal issues, vision, and foundations. Its components included reference sites (RSs) and action groups (AGs).

Reference sites were initially described as “regions, cities, integrated hospitals/care organizations that implement a comprehensive, innovation-based approach to active and healthy aging and can give evidence and concrete illustrations of their impact on the ground” (5, 6). This definition later evolved as a collaborative alliance of all stakeholders in a region, rather than a single entity organization. Therefore, RSs are ecosystems aimed at adopting and transferring creative and practicable solutions to improve the quality of life and health of older people and the whole community, increasing equity and social sustainability, and bringing together leading regional organizations committed to investing in and implementing innovation.

Action groups gathered of professionals, entrepreneurs, researchers, and experts committed to working on active and healthy aging in several areas included in its main pillars.

Since 2012, four calls for RSs have been launched. The first one (2013) emphasized key overall strategies addressing the demographic challenge in Europe. Calls opened in 2016, 2019, and 2022 adopted the following key characteristics:

- The “Quadruple Helix” model (public authorities and health and care providers/researchers/SMEs/civic society) to ensure all stakeholders have a common understanding of the organizational, technical, and financial challenges facing the region or area within health and active and healthy aging and are working collaboratively to define and implement innovative solutions and possibilities for economic growth.
- Comprehensive strategies being in place, or under development, which directed and guided policies and practices in the region, including supporting an active and healthy aging population, e.g., innovation strategies, R&D strategies, smart specialization strategies, older people strategies, education and training strategies, economic strategies, and regional development strategies.
- A strategic “whole system approach” in responding to health, societal, and economic challenges that delivered against the EIP on AHA triple win objectives.
- The degree of their alignment with the EIP on AHA through both contributions to the three EIP on AHA pillars and commitments of the EIP on AHA action plans.
- Partnerships with other regions for the transfer and exchange of good practice and/or joint working on projects to support health and care, including active and healthy aging.
- Commitment to contributing to the European evidence base demonstrating impact on outcomes for patients and service users; effectiveness of developed solutions in meeting need; and how provider organizations have adapted to deliver new services and service models.
- Evidenced impact of good practices and the degree to which smart health and care solutions for active and healthy aging have been scaled up or are being scaled up.

The fourth call for RSs (2022) included the following key criteria, together with the essential one on the “Quadruple Helix” approach:

1. Political, organizational, technological, and financial readiness.
2. Sharing learning, knowledge, and resources for innovation.
3. Contributing to European co-operation and transferability.
4. Delivering evidence of impact against the triple win approach.
5. Contributing to the European Digital Transformation of Health and Care.
6. Scale of demonstration and deployment of innovation.

This 2022 call for RSs was supported by the EC (General Directorate of Communication Networks, Content and Technology) and organized by the Reference Site Collaborative Network (RSCN). RS application assessment included the above-mentioned criteria as Phase 1 and Phase 2 of self-assessment of the maturity of the health system for active and healthy aging with a life course approach, using the tested and validated SCIROCCO tool (7–9). The SCIROCCO tool was adapted to measure the maturity of systems to address an active and healthy life in a certain territory. The tool facilitates comparison and learning, focusing on a collaborative assessment. The model consists of 12 dimensions: readiness to change, structure and governance, digital transformation, stakeholder coordination, funding, removal of inhibitors, population approach, citizen empowerment, evaluation methods, breadth of ambition, innovation management, and stakeholder’s capacity building and development. Using a restricted-access online survey tool, each dimension is evaluated using a six-position maturity scale by a group of Quadruple Helix experts.

After the first call for RSs of the EIP on AHA and following a self-assessment and a peer review process in 2013, 32 RSs from 12 member states were recognized, ranking from 1 to 3 stars. These 32 RSs collectively formed the RSCN (10, 11). The RSCN was enlarged after the 2016 call (12), with 74 RSs, and became a formal non-for-profit association under the Belgium law in 2017. The 2019 call recognized 104 RSs, a number that was reduced to 65 RSs after the last call in 2022. Currently and after this last call of 2022, the network has evolved toward addressing active and healthy living (AHL). The RSCN helps to facilitate twinning and advisory activities that help regions and organizations to better understand the local conditions that enable the successful deployment of the AHA and AHL in the European community.

In parallel, EIT Health was established in 2015 as a “knowledge and innovation community” (KIC) of the European Institute of Innovation and Technology (EIT), focusing on health and aging. The so-called “knowledge triangle” (research, business, and education) is the principle that when experts from business, research, and education work together as one, an optimal environment for innovation is created.

## 2 Policy options and implications

Andalusia, a region in the south of Spain with 8.5 million inhabitants, has been an active participant in both innovative initiatives in EIP on AHA since its beginning and later joining EIT Health. The region has been recognized as the reference site with the highest category among all the different calls for RSs. Led by the Regional Government of Andalusia, particularly by the Regional Ministry of Health, the region has been actively involved and has fulfilled the different assessment criteria. In 2013, it was recognized as a three-star RS due to its active and healthy aging and e-health strategies. In 2016, Andalusia was awarded as a four-star RS due to its excellence in adopting the Quadruple Helix approach, seeking synergies that design knowledge ecosystems with a commitment to exchange and collaboration (13, 14). In 2019, Andalusia also achieved the highest recognition as a four-star RS with special recognition of excellence, continuing the Quadruple Helix approach and contributing to the European Digital Transformation of Health and Care. The region has a formal policy commitment that sets active and healthy living as a strategic priority, several plans and strategies addressing main health and care challenges, and a priority in its smart specialization strategy (S4Andalucía).

In the 2022 call, various entities involved in the Andalusia RS covered the Quadruple Helix spectrum in which the academic world, private initiatives, the public sector, social organizations, and citizens actively participate. Among these actors, it is worth highlighting organizations dependent on the Departments of Health and Consumer Affairs and Social Inclusion, Youth, Families, and Equality, such as the Andalusian Health Service, the Andalusian Social Services, and Dependency Agency or the Andalusian School of Public Health; the Public Universities of Andalusia (Almeria, Cádiz, Córdoba, Granada, Huelva, Jaén, and Sevilla); the industry (Indra-Minsait, Fujitsu, Phillips, NTT Data, and Tunstall); and civil society such as the Andalusian Council of Official Colleges of Pharmacists, scientific societies, local social services, pharmacies, scientific societies, technology companies, active participation centers, senior associations, and patient associations. Even without a formal statement for this involvement, multilateral collaborations have been crucial in the development and implementation of innovative initiatives in the region.

Overarching regional strategies have contributed to the recognition of Andalusia as RS, such as the Andalusian Health Plan or the Andalusian Plan for the Promotion of Personal Autonomy and Prevention of Dependency, as well as several comprehensive plans (care plan, integrated care for patients with multiple chronic diseases, diabetes, oncology, palliative care, and others). In addition, Andalusia RS has a well implemented Digital Health Strategy, which includes a corporate electronic health system -Diraya-, e-prescription system, and ClicSalud+ platform for patients’ online access to their health data, as well as links with other innovation systems, Reference Sites and initiatives, such as EIT Health.

**Figure 1:**
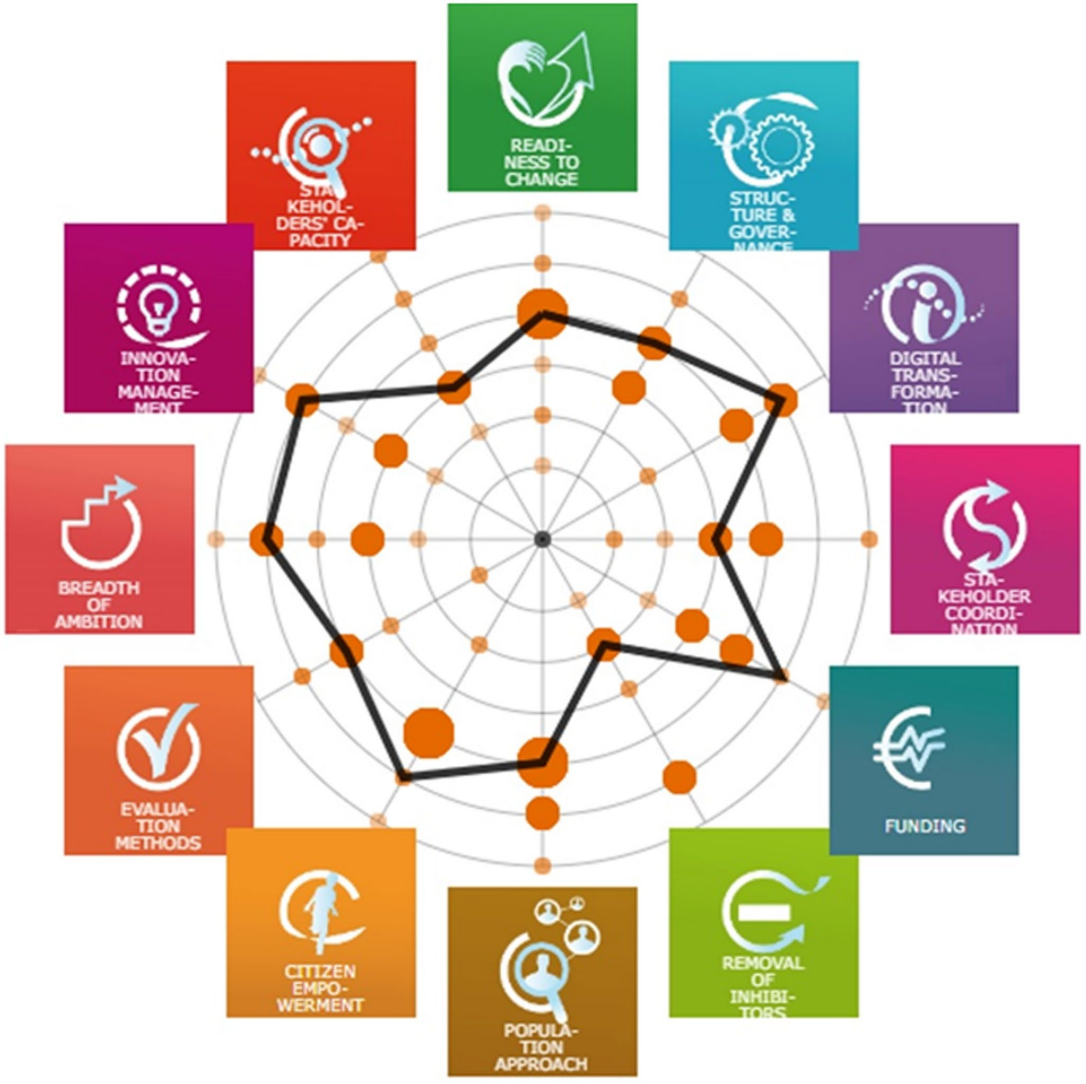
Andalusian average dimensions diagram representation of regional maturity self-assessment using the SCIROCCO tool.

All entities from the Quadruple Helix approach in Andalusia contributed to Phase 2 of the 2022 call in the self-assessment of the maturity of the health system for active and healthy aging with a life cycle approach, using the adaptation of the SCIROCCO tool specially designed for this purpose. The results of the voting and consensus were achieved through a virtual meeting on 13 September 2022. The tool offers a visualization of the results for Andalusia in a spider net diagram (Figure 1), which allows analyzing the level of consensus of the respondents, including individual scores and consensus. The number of responses was sufficient to obtain significant results. The final scores for the 12 dimensions were readiness to change: 3, structure and governance: 3, digital transformation: 4, stakeholder coordination: 2, funding: 4, removal of inhibitors: 1, population approach: 3, citizen empowerment: 4, evaluation methods: 3, breadth of ambition: 4, innovation management: 4, and stakeholder’s capacity building and development: 2.

Several innovative Andalusian practices have been shared within the RS community and are listed in the EC repository of good practices—Futurium platform**—**on active and healthy living in the digital world. These practices are as follows:

- Diraya-ClicSalud+, a corporate health information system including a shared electronic health record for each individual and secure online access to their health data.
- The population health database, curated data repository incorporating health information of all individuals with a healthcare record in the public healthcare system.
- The Andalusia Health App, to access different services and the repository of corporate apps.
- EnBuenaEdad, an online platform fostering active and healthy aging.
- Andalusian Telecare Service (ASSDA), a proactive telecare service for the prevention of loneliness in elderly and vulnerable populations.

**Figure 2:**
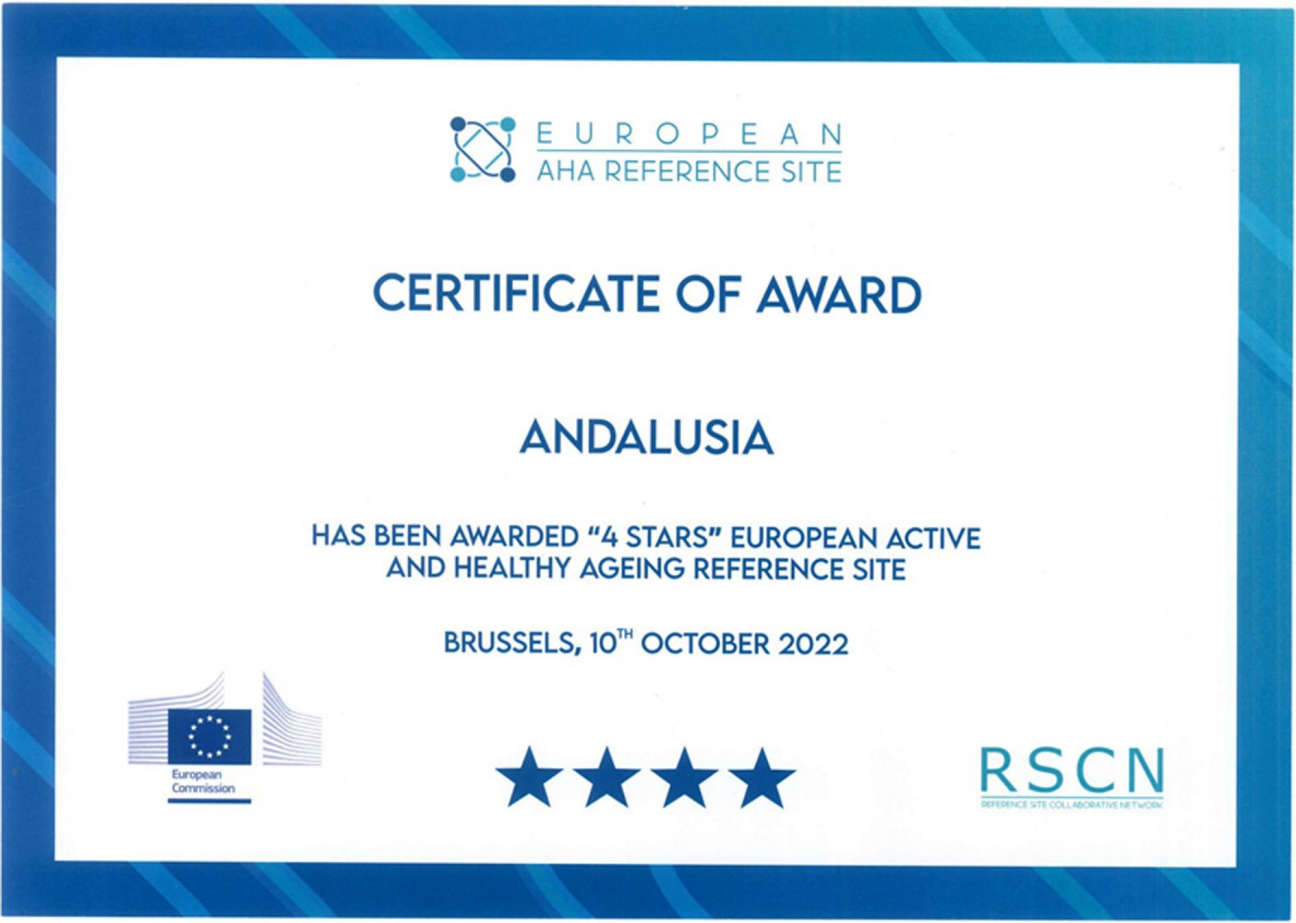
Andalusian RS Certificate of Award 4 stars.

Andalusia was once again recognized as an RS with the highest rating of four stars in the fourth call of 2022, reflected in the Certificate of Award in Figure 2.

Collaboration and exchange of good practices among RSs and participation in EU-funded projects have been an added value since the beginning of the EIP on AHA. Andalusia RS has been involved in several twinnings and study visits, strengthening synergies and facilitating the development and scaling up of innovative solutions: with the City of Zagreb RS on the Andalusian Digital Health Strategy—Diraya—and with Scotland to learn more about the Scottish “Living it Up” platform, in 2017. In 2018, a study visit to Basque Country to learn about the Basque Social-Health Strategy. During 2022, the Andalusian ClicSalud+ best practice was shared with Scotland, and the population health database was shared with Algarve (Portugal). In addition, a specific workshop “Digital Health for all” organized by Andalusia RS was held at the Committee of the Regions in Brussels on 25 October 2018 to share the priorities established in the “Communication of the European Commission for the transformation of health services in the digital single market, contributing to the empowerment of citizens and the construction of a healthier society” [COM (2018) 233 final].

It is not easy to assess the impact of the different regional policies and strategies addressing innovation in active and healthy living. Several indicators have been selected to show some results on the different dimensions of the EIP on AHA, such as the use of healthcare services, the overall health status of the population, and knowledge generation.

**Figure 3:**
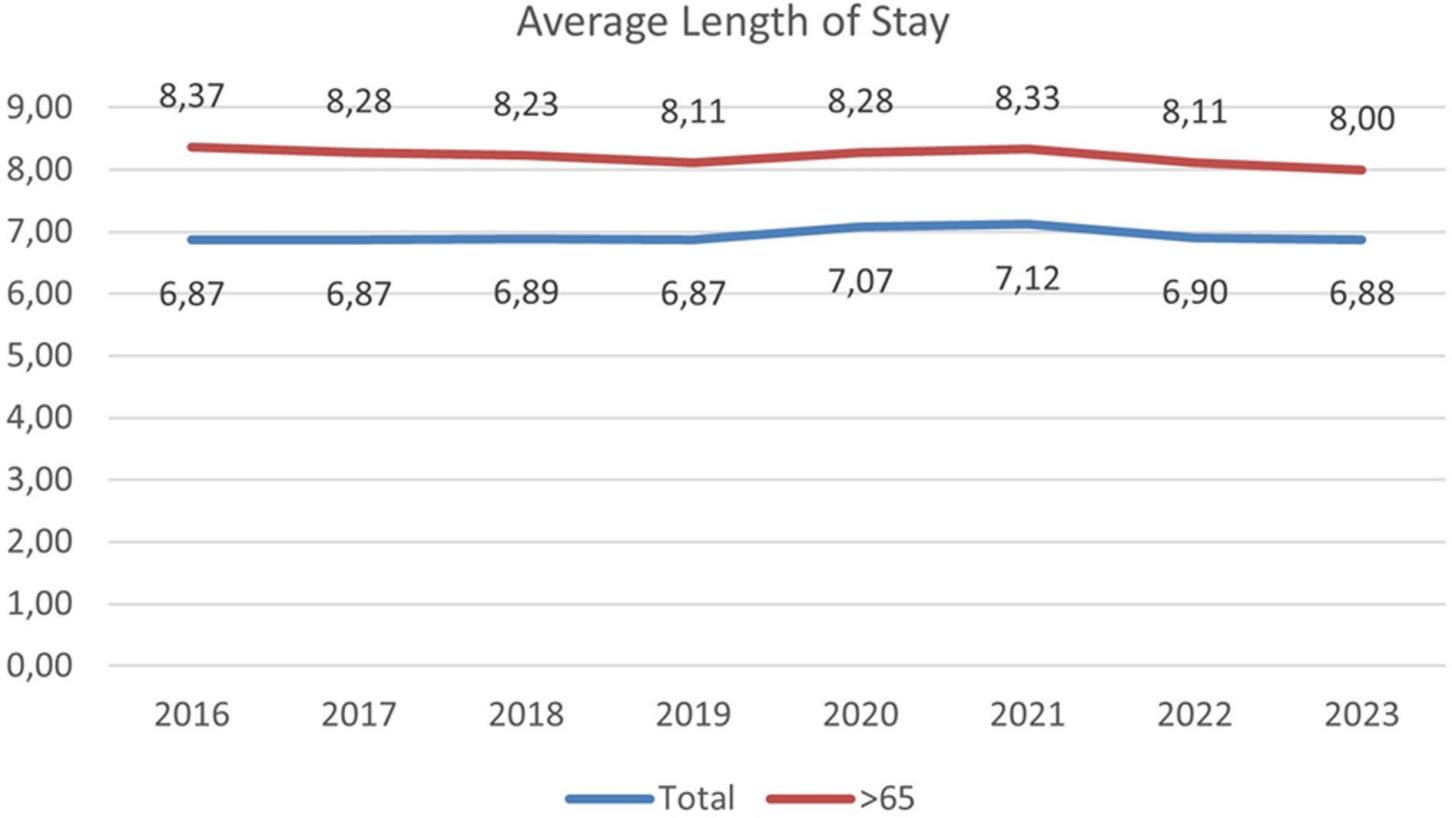
Andalusian average length of stay. Source: data from the Cartography and Statistics Institute of Andalusia.

The first selected indicator is the average length of stay, reflecting an overall proxy of healthcare service utilization and its impact on efficiency and sustainability. Figure 3 depicts its evolution from 2016 to 2023, including the average length of stay for the total inpatient population and those over the age of 65. The average length of stay remains more or less constant for the total inpatient population but decreases for those over the age of 65 except the years affected by the COVID-19 pandemic. Multiple factors can influence this trend, but shorter inpatient stays contribute to the overall sustainability of the healthcare system.

**Figure 4:**
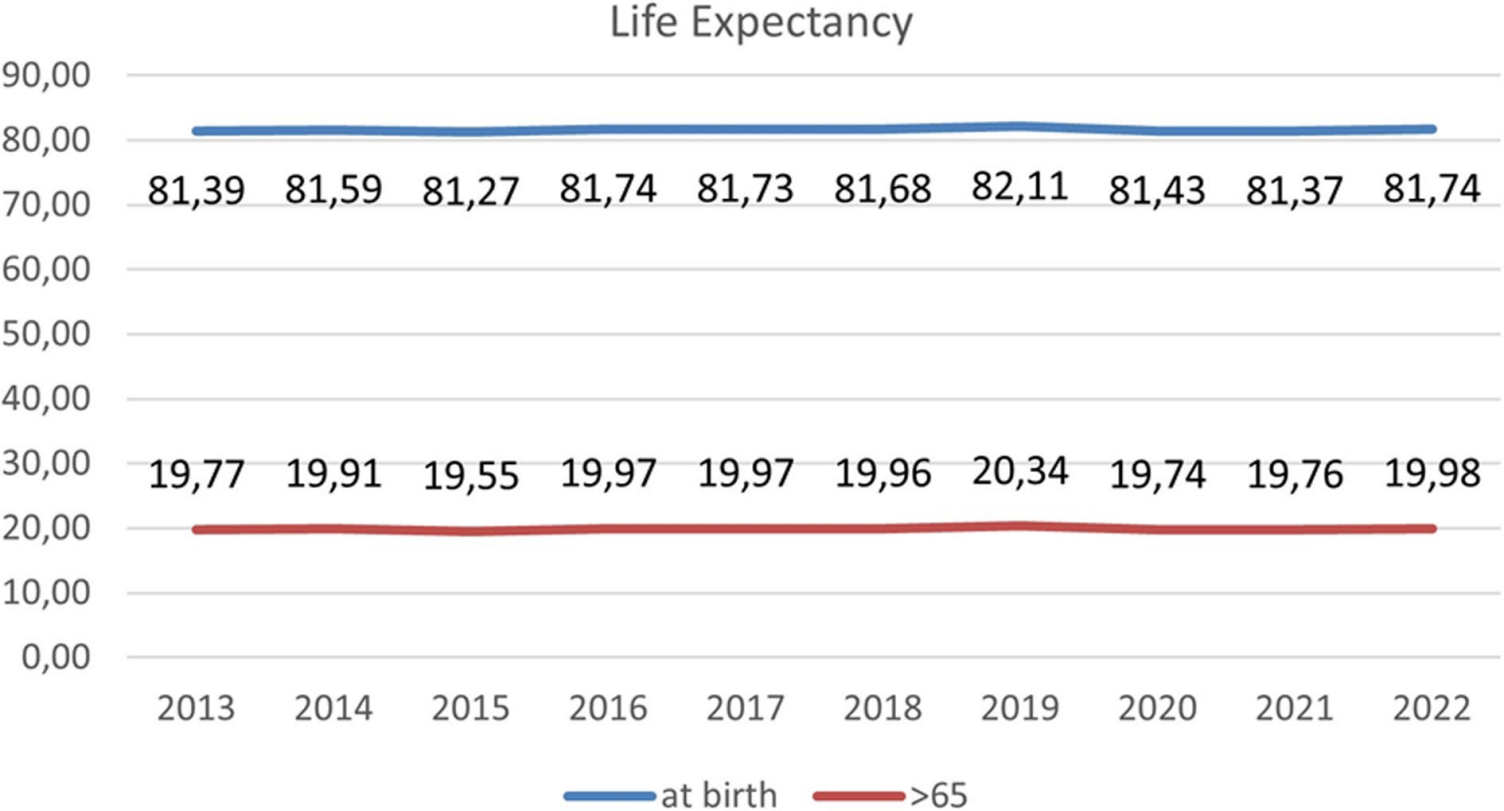
Andalusian life expectancy at birth and > 65. Source: data from the Cartography and Statistics Institute of Andalusia.

A more comprehensive indicator for assessing the overall health status of the population is life expectancy at birth and at 65 years, as depicted in Figure 4, covering from 2013 to 2022. Both indicators follow an upward trend, also affected by the years of the pandemic, showing a positive direction. Like the previous indicator, a wide variety of factors affects its evolution.

**Figure 5:**
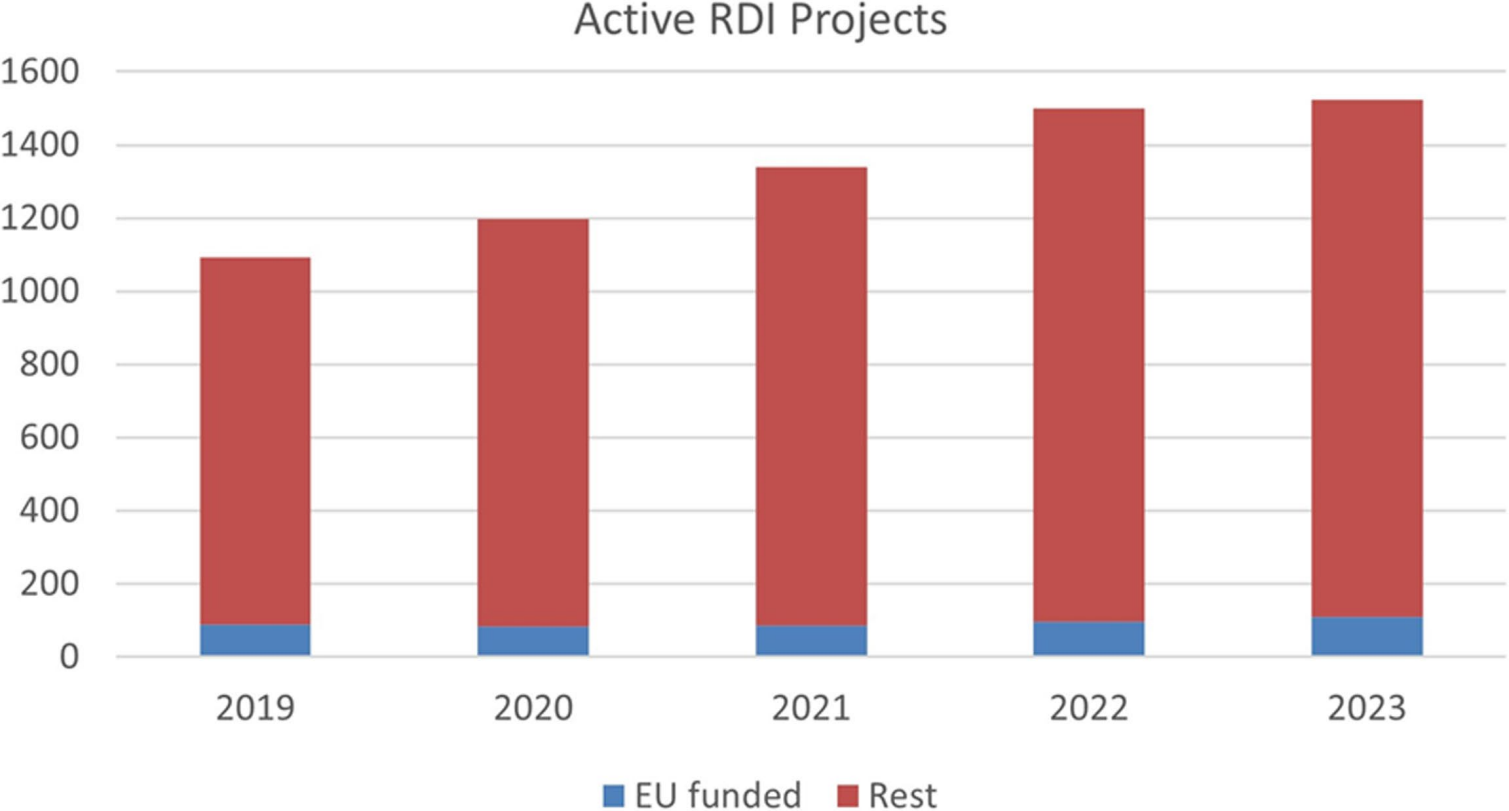
Andalusian Active Research and Innovation Projects. Source: data from the cartography and Statistics of Institute Andalusia.

Exploring the implications of the options for generating research, development, and innovation (RDI) projects active in the region between 2019 and 2023, an upward trend is clearly reflected, as depicted in Figure 5. The total number of active RDI projects has moved from 1,094 in 2019 to 1,523 in 2024, roughly a 50% increase during these 5 years. Participation in EIT Health has also fostered collaboration with businesses and higher education institutions, and several projects have been developed, which have contributed to the positive trend in this indicator.

## 3 Actionable recommendations

The commitment to improving the health of the overall population including the elderly one in the region of Andalusia has been maintained and supported by the successive governing teams, with different political parties. This continuous political engagement has remained since the beginning of the EIP on AHA and remains today and has been reflected in the overarching regional plans and strategies.

Overall positive results regarding the impact on healthcare utilization, population health status, and fostering research and innovative projects have encouraged the participation in this European initiative and the continuity of the political support to date, inspiring policymakers’ involvement, even with major changes in political leaders that have challenged this support.

Reference Sites have been most successful when they have brought together all the key stakeholders—regional government, health and care providers, industry, academia, and civil society—into a coherent partnership or ecosystem. This “Quadruple Helix” arrangement has enabled all stakeholders to be more aware of the health and care priorities, challenges, and needs. This has enabled researchers and industry to focus on more rapidly developing solutions to be tested, and where a positive evidence base is demonstrated, offering mechanisms to scale up within the region and allowing mutual learning with other initiatives and innovative ecosystems in other territories. Lacking a formal statement for the formation of the regional partnership has not limited the development of research and innovation in the region, as has been reflected in their contribution to the SCIROCCO maturity model self-assessment. Minor difficulties in understanding the model and its dimensions were overcome with the support of the technical team at the Regional Ministry of Health.

The adoption of a strategic approach within an innovative ecosystem has allowed us to focus on the benefits of innovative practices and solutions. Participation in the RSCN has contributed to the exchange of knowledge and identification and sharing of practices between RSs, enhancing the network and all RSs involved (15). Assessing the impact of these initiatives is challenging, but some positive results in several aspects have been shown. Having this focus on outcomes therefore provides a “Triple Win,” which all stakeholders have contributed to.

## 4 Conclusion

The participation of Andalusia as RS first on active and healthy aging and now on active and healthy living has strengthened the collaboration of all stakeholders involved in the formation of innovative ecosystems under the Quadruple Helix approach. Showcasing the initiatives implemented in the region and mutual learning and connections with other RSs have been facilitated by the RSCN, in which Andalusia has been involved since its creation and formal constitution as vicechair of the network.

Different actors from academia, industry, and policy, together with civil society, influence the structure and design of a knowledge ecosystem in Andalusia. Complementary effects from innovations interact and propagate the effect of overarching policies and strategies to achieve better health for the population in the region (16).

Specific tools, such as the SCIROCCO one, help regions to understand the strengths and weaknesses of their regional context and inform policymakers about possible areas for improvement and foster innovation. It can also be used to compare the level of progress in a region before and after introducing reforms or innovations. Comparisons with other territories are also possible, and opportunities for exchange and shared learning can arise, strengthening collaborations in the field of health and care innovations. Minor difficulties in the understanding of its different dimensions do not limit its usage.
